# Identifying Stakeholder Values for an eHealth Intervention to Facilitate Home-Based Geriatric Rehabilitation: A Qualitative Multi-Method Approach

**DOI:** 10.1177/00469580251347139

**Published:** 2025-07-11

**Authors:** Michael Bernardus Gustaaf Zonneveld, Margriet Christine Pol, Wilco Pieter Achterberg, Eléonore Françoise van Dam van Isselt

**Affiliations:** 1 Leiden University Medical Center, The Netherlands; 2 Amsterdam Public Health Research Institute, Amsterdam University Medical Center, Vrije Universiteit Amsterdam, The Netherlands; 3 Amsterdam University of Applied Sciences, The Netherlands

**Keywords:** older adults, telehealth, telerehabilitation, co-creation, design thinking, outpatient rehabilitation, digital health

## Abstract

Despite the existing need for an integrated eHealth intervention for home-based geriatric rehabilitation, it has never, to our knowledge, been developed through co-creation with the stakeholders. This study aims to identify values (based on stakeholders’ needs and preferences) regarding an eHealth intervention that fits the context of home-based geriatric rehabilitation. The multi-method qualitative design of this study comprised multiple qualitative methods (focus groups, interviews, and brainstorming sessions) and a survey. An agile science approach allowed for iterative data collection and analysis until an optimal fit was achieved. Recommendations from The Center of eHealth Research and Disease Management (CeHRes) Roadmap were used. Fourteen key stakeholders were identified, seven of which were represented in a project team that co-created the steps of this research. Based on the results of the qualitative methods, 15 eHealth attributes were formulated that captured the needs or preferences of the stakeholders. These resulted in the formulation of seven stakeholder values: (1) Fit with the patient’s digital skills and needs, (2) Blended care, (3) Personalized, (4) Safety at home, (5) Affordability, (6) Support, and (7) Privacy. Co-creation with stakeholders is necessary for the development of eHealth interventions in home-based geriatric rehabilitation. The most prominent key stakeholder is the patient. eHealth should match with the digital skills of the patient and preferably be delivered as blended care. The formulated values should be incorporated in future development of eHealth interventions.

**Highlights**

● Co-creating with key stakeholders is essential for the development of eHealth interventions that fit the context of home-based geriatric rehabilitation.

● There is a need for eHealth to fit the digital skills and needs of the patient.

● eHealth should be delivered as blended care.

## Introduction

Geriatric rehabilitation is important for older adults, as they are at high risk for negative outcomes after hospitalization, such as losing the ability to live independently.^1^ To overcome these negative outcomes, geriatric rehabilitation (post-acute restorative care after hospital admission) aims “to achieve a new balance, which implies a higher degree of dependency while preserving autonomy, and self-management as much as possible.”^2^

Geriatric rehabilitation is generally offered as an inpatient care trajectory in for example a skilled nursing facility. However, it can also be provided as a home-based service.^2^ In this study, home-based geriatric rehabilitation is defined as the delivery of multidisciplinary geriatric rehabilitation in the patient’s home environment. Research indicates that home-based geriatric rehabilitation can be as effective as inpatient geriatric rehabilitation, but may offer several advantages, such as improved functional performance.^3,4^ However, home-based geriatric rehabilitation is only provided on a limited scale in the Netherlands.^5-7^

Several barriers have been identified that explain why so little home-based geriatric rehabilitation is provided. The barriers can be related to the patient (a lack of self-management skills, which could reduce their improvement potential), to the professional (lack of consensus on the advantages of home-based geriatric rehabilitation, which could cause the care pathway to remain inside the rehabilitation center), and organizational (lack of a proper financial structure to support traveling times between patients).^8^

The use of eHealth is expected to be valuable in overcoming some of these barriers. eHealth can be an effective way of delivering treatment and has the potential to improve the efficiency of care.^3,9^ Examples of eHealth interventions include mobile applications, video communication, and sensor technology. It may provide valuable monitoring data and telerehabilitation, while the patients are at home, for both the older adult, and the professional.^4^

eHealth is only used to a limited extent by healthcare professionals and has not yet been integrated into geriatric rehabilitation care pathways.^10^ Without this integration it does not become a part of standard care in The Netherlands. Therefore, eHealth should be developed, implemented, and evaluated in a participatory process together with all stakeholders, and integrated into the care process.^11-13^ Despite the existing need for an integrated eHealth intervention for home-based geriatric rehabilitation,^14^ it has never, to our knowledge, been developed through co-creation with the stakeholders. The first step in the development of an eHealth intervention, before designing the intervention itself, is to identify and prioritize the stakeholder values and translate them into specific requirements.^15^

The current study therefore aims to identify and prioritize the stakeholders’ values for an eHealth intervention and translate them into specific requirements that fit the home-based geriatric rehabilitation context.

## Method

### Design

**Figure 1.**
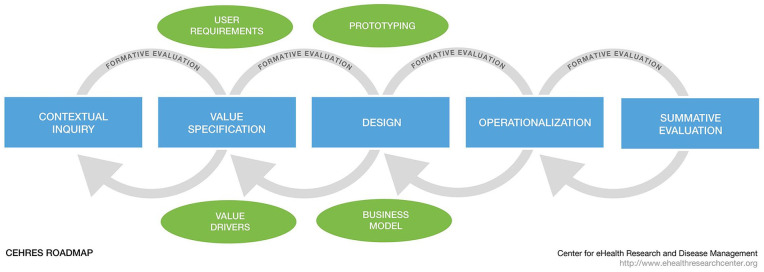
The CeHRes Roadmap (the Centre for eHealth Research Roadmap).

This study used a multi-method qualitative design, comprising multiple qualitative methods with an agile science approach in which data collection and analysis were conducted in parallel and iteratively until an optimal fit to the context was achieved.^16^ Determination of the stakeholder values that fit the context was supported by the recommendations of the Center of eHealth Research and Disease Management Roadmap (CeHRes Roadmap; Figure 1),^16^ a framework for developing and implementing eHealth interventions in complex contexts.^11^ The framework consists of 5 phases. This study focused on phases 1: contextual inquiry, and 2: value specification. All study procedures received institutional ethics approval from the medical ethical committee of the Leiden University Medical Center. The reporting of this study is in accordance with the COREQ guidelines.^17^

### Contextual Inquiry

The contextual inquiry aims to (1) identify and analyze all relevant stakeholders, and (2) describe the current situation of the use of eHealth in home-based geriatric rehabilitation in a rehabilitation center in The Netherlands, and also identify the strong and weak points in order to find out if and how eHealth can be integrated.

Stakeholders were identified using stakeholder salience approach by Mitchell et al.^18^ Following the step-by-step guide for stakeholder involvement of van Woezik et al.^13^ The identified stakeholders have to determine which stakeholders are to be considered key stakeholders.^18^

A multidisciplinary project team was formed with the key stakeholders. This project team was tasked with continuous formative evaluation of the outcomes obtained so far and whether these are taken into account in the next steps,^15^ and were providing direction for the regular meetings.^19,20^

Several methods were used to gain an understanding of the current situation of eHealth use in geriatric rehabilitation. Semi-structured interviews were conducted with older adults in geriatric rehabilitation to investigate their support and enthusiasm for the use of eHealth in home-based geriatric rehabilitation, and to identify areas of improvement. These interviews were conducted by a male researcher MZ, who is trained in, and experienced with, interviewing. There was no relationship established with the participants beforehand. Aim of the study was explained in the information letter beforehand, and clarified before signing the informed consent forms. Participants’ demographic data (gender, age, marital status) and digital skill level were collected. The digital skill level was measured using the Quickscan developed by Pharos.^21^ The interviews were conducted using an interview guide. This guide was pilot tested beforehand and included questions about previous experiences with eHealth, their ideas regarding eHealth for home-based geriatric rehabilitation, which device they would prefer, and how eHealth could improve home-based geriatric rehabilitation. Furthermore, they were asked their opinion on different types of eHealth. The full interview guide is presented in Supplemental 1.

Focus groups were held with healthcare professionals to determine the support and enthusiasm for the use of eHealth and to identify their ideas about potential ways of providing home-based geriatric rehabilitation with eHealth. The focus groups were led by a male researcher MZ and aided by female researchers DB (focus group 1) and LO (focus group 2). All researchers have had training and experience with the focus group methodology. Participants of the focus groups were indirect colleagues of researcher MZ, who works for the same organization. The central question was: Which hindering and promoting factors are you experiencing in implementing eHealth in home-based geriatric rehabilitation? To also gain insight into the values of eHealth and the functionalities needed according to stakeholders, a second question was asked: What are the most suitable kinds of eHealth applications in the context of providing home-based geriatric rehabilitation? See complete topic guide Supplemental 2.

Participants were selected based on their replies to a first information letter that was send to all patients and healthcare professionals in the rehabilitation center. After replies of interest a full information letter was send, they were able to ask questions before signing an informed consent form.

### Value Specification

The value specification phase builds on the outcomes of the contextual inquiry and aims to determine the values of eHealth in the context according to the previously identified key stakeholders.^22^ Values are therefore a specification of what the key stakeholders want to achieve with the eHealth technology and what the values for all involved stakeholders are, not just for the end users.^19,23^

The information obtained from the interviews and the focus groups was shared with the project team before multiple individual brainstorming sessions were held to generate ideas for what eHealth technology fits the context.^24,25^ The ideas were selected by consensus and described in more detail in several scenarios. They were presented to the stakeholders in a web-based questionnaire intended to identify their preferences and values. They rated each scenario on a 10-point Likert scale and had the option to provide feedback for improvements.

eHealth attributes were formulated based on the results of all the previously described methods, with each attribute intended to capture the needs or preferences of the stakeholders. The attributes were checked with the project team before being categorized. From the categorized attributes, related values were formulated that stated what eHealth for home-based geriatric rehabilitation should achieve, improve, or add according to the stakeholders.^15,26^ The values were discussed in the project and research teams and minor adjustments were made to the wording.

### Data Analysis

The quantitative data from the stakeholder analysis survey was analyzed using descriptive statistics. To be assigned the trait, the stakeholder had to receive the trait by at least 50% of the participants.

For the interviews and focus groups, a thematic analysis with a latent approach and open coding was used to stay as true to the participants’ expressions as possible.^27^ Field notes were made and the audio recordings were transcribed verbatim and coded independently by 2 researchers (MZ and MP) using Atlas.ti.^28^ Transcripts were not returned to participants for comments before coding. Consensus on codes was reached with the research team before grouping them into categories and subsequently into themes. The coding focused mainly on the current situation of eHealth in home-based geriatric rehabilitation, but values and technological requirements were also coded. Participants did not provide feedback on the findings.

All preliminary results were presented to the project team before brainstorming about possible scenarios. The Likert scale data from the web-based questionnaire were analyzed descriptively with SPSS, while the open-ended questions of each scenario were analyzed qualitatively using the codes from the interviews and focus groups.

Based on all the results, the data were synthesized into the formulation of eHealth attributes that capture the needs or preferences of the stakeholders. The project team checked the attributes prior to categorization and formulated and checked the values after categorization. Adjustments were made to the wording before finalizing the values.

## Results

### Contextual Inquiry

#### Stakeholder Identification & Analysis

An initial list of 25 stakeholders was obtained through desk research, scanning both scientific and gray literature. This list was reviewed by 2 field experts for validation, and 1 stakeholder was added to the list. After the validation, 4 different stakeholders were interviewed to identify any missing stakeholders. Multiple stakeholders were added, resulting in a total of 37 stakeholders. These 37 were validated again by a third field expert.

The stakeholder analysis survey was answered by 21 stakeholders from 10 different professional backgrounds. Of the 37 stakeholders, 14 were determined to be key stakeholders. The patient was found to be the only definitive stakeholder. The results for all 37 stakeholders can be found in Supplemental 3.

##### Formation of the Project Team

The project team was formed immediately after the stakeholder analysis and included 3 participants from a local elderly council, a physiotherapist, an elderly care physician with expertise in rehabilitation, a policy maker, a manager, a representative from an insurance company, and the principal investigator. The project team met a total of 5 times over the course of 7 months and discussed the preliminary results of the contextual inquiry and value specification. In each meeting, the future direction of the study was discussed.

### Current Situation of the Use of eHealth Home-Based in Geriatric Rehabilitation

#### Interviews

Eight patients participated, ranging in age from 66 to 86 years old. Seven interviews were held in the rehabilitation center and 1 at home the day after discharge. There were 7 women and 1 man, with varying digital health skills (1 beginner, 2 average, 5 experienced) for a full overview of participants see Supplemental 4. One participant had her partner present during the interview. The duration of the interviews were between 30 and 45 min. Open coding of the interviews resulted in 647 unique codes. These codes were grouped into 53 categories and discussed with the project- and research team, which resulted in 3 themes: (I) eHealth should fit the digital skills and needs of the end user, (II) the wish to return home, (III) the need for support.

I) eHealth should fit the digital skills and needs of the end user

Four participants specifically mentioned the importance of digital skills to be able to use eHealth. Adopting eHealth could be problematic for persons with an insufficient digital skill level.

Participant E: “I find it very difficult. For me it would be a problem. Because I am not digital at all and I’m very ignorant on the topic, so yes, for me that would be quite a problem. Definitely.”

Furthermore, the type of eHealth to be introduced should be easy to use and tailored to each older adult’s needs and interests. Only 1 of the participants had experience using eHealth, but 7 of the 8 participants use their digital devices regularly. Each has their own reasons and motivation for continuing to use them, such as searching for information, video calling relatives, social media, and playing (online) games. Without the proper motivation or interest in adopting the technology as part of the rehabilitation process, the use of eHealth in home-based geriatric rehabilitation will not have the desired effects.

II) The wish to return home

Five participants expressed the wish to return home as soon as possible. Especially if they could continue their exercise program and work on their goals using eHealth. However, due to their lack of experience with eHealth, they were not sure how they could benefit. Two participants mentioned a willingness to try eHealth if it is safe to return home and they can stay in contact with professionals. During rehabilitation, they received physical and emotional support from both the professionals and other patients, and they would like to maintain this social aspect. One of them even said that digital healthcare could help to prevent social isolation if they can stay in touch with other patients or healthcare professionals.

The prospect that eHealth will be implemented in the future and it could become mandatory scared some of the participants. They are afraid of losing the social aspect and feeling less safe during therapy. In light of the importance of support, 5 participants mentioned there should always be a combination of eHealth and in-person therapy.

Participant A: “I think, if you do it correctly, digital therapy, there should still always be a moment that a real physiotherapist will check that you are doing the exercises the right way.”

III) The need for support

According to the participants, many kinds of support will be needed. Six of the participants expressed that they had family and friends to help them use eHealth and they emphasized the need to have them around to help, for example, with technological issues. One participant mentioned that she doesn’t want to burden her daughter with the help that she needs and so she doesn’t ask.

Participant H: “And then I look at my daughter and I think ‘you know how to do it too.’ You know. It’s so darn easy for them. ‘Yes Mom,’ she says, ‘but I also had to learn. You know. Because in the beginning I didn’t do it either, I also learned from my daughter,’ she says.”

#### Focus Groups

Two separate 90-min focus groups were held in the workplace, with 3 and 4 participants, respectively, from 5 different professional backgrounds (Supplemental 4). Lessons learned from the first focus group were incorporated in the second session.

The opinions on the most suitable eHealth application in the context of providing home-based geriatric rehabilitation served as input for the project teams’ generation of ideas in the next phase. The hindering and promoting factors influencing the implementation of eHealth in home-based geriatric rehabilitation, according to the healthcare professionals, can be categorized on 3 levels: (I) organization, (II) professionals, (III) patients.

I) Organization

The healthcare professionals mentioned the interest and curiosity on the organizational level on eHealth as a promoting factor. There should be a budget not only for a 1-time purchase, but also for the licensing in the following years. In addition to funding the eHealth intervention courses, time is needed to get accustomed to the specific interventions. The organization needs to keep up to date with privacy legislation and ensure the licensed eHealth conforms to this legislation.

Participant K: “The employer has room to investigate it as well as money or budget to give it a try. So that, there’s no real resistance to starting something new, I think that’s great. So there is space; it’s just: how do we use it.”

II) Professionals

The professionals are motivated and willing to try new things. According to them, having the motivation will help the implementation of eHealth. However, they say that they need time to familiarize themselves with an eHealth application or intervention before they can use it in practice. They need sufficient knowledge and digital skills, which is important to motivate the patient. Some professionals also mentioned a feeling of resistance to outsourcing part of the therapy to the eHealth intervention, a feeling of loss of control in personalized treatment.

Participant L: “Yes, you need to motivate people don’t you, and let them try things. Show that it works and it supports their treatment. And if you as the professional dare to use it, well, maybe you can convey that to your patients.”

III) Patients

Healthcare professionals in both groups mentioned the need for a sufficient digital skill level before the patient is able to start using eHealth. Another factor is the presence of an informal caregiver who can help if necessary.

Participant N: “His children, many from his network, were far away in Canada, so he was determined to become digitally skilled. That is, of course, intrinsic motivation. And, well, if you get everything handed to you on paper, you are less motivated.”

### Value Specification

#### Identifying Needs and Preferences

Based on the preliminary results of the interviews and focus groups, the project team started brainstorming sessions to generate ideas for a future scenario. The main goal of this scenario was that eHealth is used to continue rehabilitation at home in a safe and guided manner, and the patient can continue training independently.

The ideas were discussed, and 4 scenarios emerged and were described in more detail:

1. Synchronous videoconferencing: the patient has appointments with the doctor and/or healthcare professionals from the rehabilitation center. These appointments take place in a live videoconference and the therapy takes place in that moment.
2. Asynchronous videoconferencing: the patient has been doing the exercises during the week, and during the live videoconference it is evaluated?. The focus is on reflection, stimulation and coaching.
3. Instructional videos: the patient has specific videos with exercises that he/she should perform. With an exercise program, they will know when and how many of each exercise they should do.
4. Website with instructions and information: with a personal account where only necessary information is visible at the right time for the right person.

The 4 scenarios that emerged from the brainstorming session were included in a web-based questionnaire that aimed to identify the stakeholders’ preferences. Over the course of 2 months a total of 27 stakeholders with 8 different backgrounds responded. The median scores on the 10-point Likert scale and the range are shown in Table 1.

**Table 1.** Stakeholders Who Answered the Questionnaire.

Stakeholders	N	Scenario 1, *M* (min-max)	Scenario 2, *M* (min-max)	Scenario 3, *M* (min-max)	Scenario 4, *M* (min-max)
Stakeholders (total)	27	5 (0-9)	6 (0-9)	6 (0.5-10)	7 (0-10)
Physiotherapist	8	4.5 (0-7)	5.5 (0.5-7)	8 (4.5-10)	7 (0-8.5)
Occupational therapist	4	3.5 (2-4.5)	8 (6-8)	4.25 (1-8)	7.5 (1-8)
Speech therapist	1	6	4	7	7
Dietician	4	7 (0-9)	4.5 (0.5-9)	3.25 (0.5-10)	4.75 (0.5-10)
Psychologist	3	5 (3.5-6)	6 (6-7)	5 (1-7)	4.5 (3-7)
Social worker	1	1	0.5	1	1
Nurse	1	6	6.5	6	6
ICT department	2	7.75 (7-8.5)	7 (6-8)	6.5 (5-8)	2.75 (0.5-5)
Other	3	2 (2-9)	6 (0-7.5)	5 (5-9)	5 (3-7)

The scores given by the stakeholders vary considerably. Both within stakeholder groups and between stakeholders. Looking at the heterogeneity of the scores, there does not seems to be a single scenario that all the stakeholders preferred. However, the answers to the open-ended questions about the positive and negative aspects of the scenario and suggestions for improving the scenario provide input for the values and attributes.

The most important aspects of eHealth use that are considered positive are the possibility to increase the number of contact moments while being more time efficient. This time efficiency is mainly related to travel time for the patient or the professional. For scenarios 3 and 4, it is the possibility to reread or rewatch any of the instructions given that is considered positive.

Aspects deemed negative are the lack of control to ensure safety during therapy and that hands-on therapy is not possible when using eHealth. The lack of personal contact is also seen as affecting the therapeutic relationship.

The suggestion made by 13 stakeholders is to create blended care by combining the eHealth scenario with in-person therapy and, in some cases, maybe combine the different eHealth scenarios into a new scenario such as a website with instructions and videos (scenarios 3 + 4).

### Needs and Preferences Derived From the Interviews and Focus Groups

During the interviews, 3 people mentioned that the cost of the technology would be a barrier for them if they had to pay for it themselves. Without reimbursement from the insurance company they would not start using it. Affordability was also a topic in the focus groups due to the limited budget within the organization for all the innovation wishes. Unfortunately, desk research showed that organizations have to bear the costs of their technologies and it must be funded from the regular reimbursement they receive from the insurance company.^29^

#### Value Formulation

Based on all the previous findings, 15 eHealth attributes were formulated, capturing the needs, or preferences of the stakeholders. These attributes were checked with the project team before being categorized, and accompanying values were formulated, indicating what eHealth for home-based geriatric rehabilitation should achieve, improve, or add, according to the stakeholders. This resulted in a final set of 7 values: (1) Fit with the digital skills and needs of the patient; important for the use of eHealth is that it matches or can be adapted to the patient’s digital skill level and physical abilities. (2) Blended care; eHealth should never replace face-to-face therapy but can be used in parallel if both parties agree. (3) Personalized; when providing an eHealth intervention, the approach should be personalized, individual goals have to be set and there is no standard platform for all patients. (4) Safety at home; a safe home environment is needed to continue treatment. (5) Affordability; all eHealth interventions come with costs. It is important for both the patient and the geriatric rehabilitation center that the costs of eHealth are low or reimbursed by the insurance company. (6) Support; when technological problems arise, support or help from others may be needed. This can be support from an informal caregiver, a healthcare professional, or an official help desk. (7) Privacy; the entire process of home-based geriatric rehabilitation should remain in compliance with privacy legislation.

The values were prioritized based on their foundation of results from the previous methodologies. Supplemental 5 provides a complete overview of how the values were built from their respective attributes and foundations.

## Discussion

This study aimed to identify and prioritize the stakeholders’ values for an eHealth intervention and translate them into specific requirements that fit the home-based geriatric rehabilitation context.

Fourteen key stakeholders were identified, 7 of which were represented in the project team that co-created the steps of this research. The combination of methods used led to the formulation of 7 stakeholder values: (1) Fit with the digital skills and needs of the patient, (2) Blended care, (3) Personalized, (4) Safety at home, (5) Affordability, (6) Support, and (7) Privacy. These values were based on the description of 4 possible scenarios: (1) Synchronous videoconferencing, (2) Asynchronous videoconferencing, (3) Instruction videos, and (4) Website with instructions and information.

In this study the importance of the patient perspective was expressed by a mixed group of stakeholders, which is in line with the geriatric rehabilitation aim to preserve the patient’s autonomy and self-management as much as possible.^2^ All stakeholders in this study were selected by the stakeholders themselves, as the literature does not provide a ready-made list of stakeholders to include.^13^ To our knowledge, this is the first study in the geriatric rehabilitation field to perform such an in-depth stakeholder analysis. Stakeholder identification has been done in the field of mental health care and patients and healthcare professionals were included, but they were not analyzed before defining them as key stakeholders.^12^ Nevertheless, all stakeholder perspectives have to be taken into consideration when integrating an eHealth intervention, as the dominance of 1 perspective can lead to overlooking the needs of other stakeholders.^30,31^ In the current study, not all healthcare professionals who could be expected to be key stakeholders when looking at geriatric rehabilitation were found to be so for home-based geriatric rehabilitation. For example the occupational therapist, who plays an essential role in discharge and continued rehabilitation at home is included, but an elderly care physician did not reach a consensus.^32,33^

The main stakeholder value identified in this study, “to fit the digital skills and needs of the patient,” is in line with the global strategy of the World Health Organization.^34^ Geriatric rehabilitation does not aim to improve the digital skills of older adults, but rather has to match the eHealth intervention to the person’s digital skill level and their rehabilitation personal needs. This matching should be done in spite of the extreme heterogeneity in the experiences and backgrounds of older adults^35^ and certain age-related barriers, such as reduced vision and dexterity issues resulting in challenges with touch screens, or cognitive impairments.^36,37^ Especially for patients with cognitive impairments, the use of eHealth can be complex, according to healthcare professionals.^10^ The general rule of thumb is to keep it simple and to not overcomplicate.^3,35,37^ Nevertheless, healthcare professionals are reluctant to start an eHealth intervention because they tend to consider older adults as being less digitally skilled.^10^ The self-ageist attitudes of older adults may reinforce this stereotype of them^36^ This was also seen in the interviews, where participants said they were not digitally skilled enough, even though they mentioned using digital devices on a daily basis.

Patients want to be sure that they will return home to a safe environment and know that they are ready for it, especially if they have to use eHealth. This is in line with the study by Lubbe et al who found that discharge home is associated with a feeling of not being ready to continue rehabilitation in the home environment.^38^ Patients can be prepared for home-based geriatric rehabilitation by working toward relevant participation goals that are continued in the home environment^38-40^ with eHealth that is aligned with these goals.^41^ Healthcare professionals want to ensure safety during therapy, and this is not possible if they are not present. However, safety concerns, such as avoiding falls, have led to the implementation of falls prevention measures that inhibit mobility^42^ and thus undermine the rehabilitation process and the results on the participation goals.

The blended care value implies that eHealth in home-based geriatric rehabilitation should never completely replace face-to-face therapy, which is in line with previous studies.^3^ Blended care can be as effective as regular intervention, especially when it is offered in a simple way.^43^ However, in practice, the acceptance of eHealth by healthcare professionals depends on prior experience, knowing the added value, and social support from colleagues.^10^ The focus groups in the current study made it clear that the participating healthcare professionals had limited experience with eHealth. They did not yet see the full potential and expressed the concern that eHealth would completely replace their therapy. This is in line with a national eHealth monitor, which shows about half of the healthcare professionals being more reluctant to use eHealth.^44^ It is, however, the combination of personal contact and the therapeutic relationship that makes blended care so effective.^38^

The 4 described scenarios, together with the formulated stakeholder values, form the basis for the future development of an eHealth intervention for home-based geriatric rehabilitation. Gamble et al found a clear split in the evidence between synchronous and asynchronous videoconferencing, with studies most commonly using synchronous videoconferencing.^45^ Synchronous videoconferencing is arguably closer to face-to-face therapy, but is less flexible.^46,47^ The results for the scenarios described in this study did not clearly favor 1 over the other, which may also be a reason to combine multiple technologies within an intervention. Following the CeHRes Roadmap, the scenario design needs to be further specified with the stakeholders, especially with regard to the outcome of participation.^3,48^

This study shows that co-creation with key stakeholders is feasible in the context of geriatric rehabilitation. However, co-creation is not without difficulties. Although a higher level of response was anticipated, only 21 persons responded to the stakeholder analysis survey, which was judged to be difficult to understand. The theory of Mitchell et al is based on management theory^18^ and it proved to be challenging to translate their definitions to this setting. For that reason, the results of the stakeholder analysis may not be a true representation of the stakeholders.

A limitation of this study was the method of convenience sampling for the interviews and focus groups. Since no sample size calculation was done beforehand it is unsure whether enough participants were included in the study.

Another strength is the agile approach. This approach ensured the use of the most appropriate method for each of the questions that needed to be answered in order to continue formulating the values.^15^ However, a study continuing the CeHRes Roadmap into the design and operationalization phases to co-create with a larger group of stakeholders is necessary before eHealth can be integrated in the care pathways of home-based geriatric rehabilitation. The scenarios and values found in the present study should be used as a foundation for the design phase, with a specific focus on participation outcomes.

## Conclusion

Co-creating with identified, and systematically analyzed key stakeholders is essential for the development of an eHealth intervention that fits home-based geriatric rehabilitation context. The most important stakeholder, the patient, should be involved throughout the process. Seven stakeholder values were formulated, highlighting the need for eHealth to fit the digital skills and needs of the patient, and the preference to be delivered as blended care. Patients are open to the use of eHealth in home-based geriatric rehabilitation provided it is safe. Future research should evaluate and integrate these values and possible scenarios into the design process for delivering eHealth in home-based geriatric rehabilitation. As well as a study to the digital skill level of the patient and aligning eHealth interventions with their respective level.

## Supplemental Material

sj-docx-2-inq-10.1177_00469580251347139 – Supplemental material for Identifying Stakeholder Values for an eHealth Intervention to Facilitate Home-Based Geriatric Rehabilitation: A Qualitative Multi-Method Approach Supplemental material, sj-docx-2-inq-10.1177_00469580251347139 for Identifying Stakeholder Values for an eHealth Intervention to Facilitate Home-Based Geriatric Rehabilitation: A Qualitative Multi-Method Approach by Michael Bernardus Gustaaf Zonneveld, Margriet Christine Pol, Wilco Pieter Achterberg and Eléonore Françoise van Dam van Isselt in INQUIRY: The Journal of Health Care Organization, Provision, and Financing

sj-pdf-1-inq-10.1177_00469580251347139 – Supplemental material for Identifying Stakeholder Values for an eHealth Intervention to Facilitate Home-Based Geriatric Rehabilitation: A Qualitative Multi-Method Approach Supplemental material, sj-pdf-1-inq-10.1177_00469580251347139 for Identifying Stakeholder Values for an eHealth Intervention to Facilitate Home-Based Geriatric Rehabilitation: A Qualitative Multi-Method Approach by Michael Bernardus Gustaaf Zonneveld, Margriet Christine Pol, Wilco Pieter Achterberg and Eléonore Françoise van Dam van Isselt in INQUIRY: The Journal of Health Care Organization, Provision, and Financing
