# Supplementary material for: Identifying Stakeholder Values for an eHealth Intervention to Facilitate Home-Based Geriatric Rehabilitation: A Qualitative Multi-Method Approach
Source: Inquiry. 2025 Jul 11;62:00469580251347139. doi: 10.1177/00469580251347139 (PMC12254665; doi:10.1177/00469580251347139)
Supplement: sj-docx-2-inq-10.1177_00469580251347139 – Supplemental material for Identifying Stakeholder Values for an eHealth Intervention to Facilitate Home-Based Geriatric Rehabilitation: A Qualitative Multi-Method Approach [file sj-docx-2-inq-10.1177_00469580251347139.docx]

**Supplement 1: Interview scheme**

Questions:

- What is your experience with geriatric rehabilitation?
- Do you use a smartphone? Or a computer/ laptop?
- What do you use it for?
- Did you ever receive care with the aid of a tablet or iPad?
- What was your opinion on it?
- What do you think about when you hear eHealth? Or digital care?

*A definition of eHealth was given together with some examples aided by pictures as seen below.*

- What could you use for rehabilitation at home?
- What device would you like to use?
- When would you choose to use digital care for rehabilitation at home?
- How could digital care improve rehabilitation at home?
- What is your opinion on the use of digital care?

Photo’s:

| Videocall  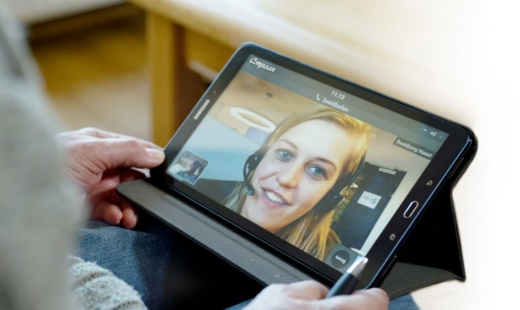 | Self monitoring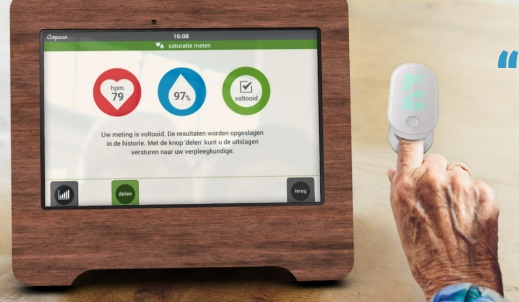 |
| --- | --- |
| Training program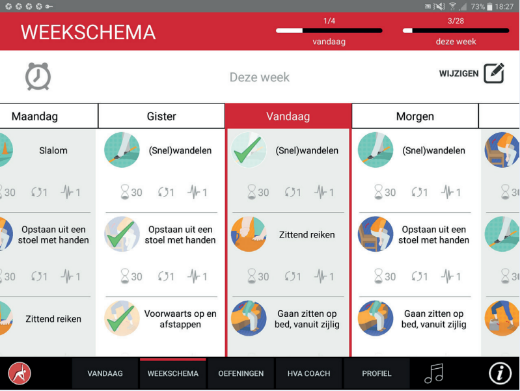 | Exergaming  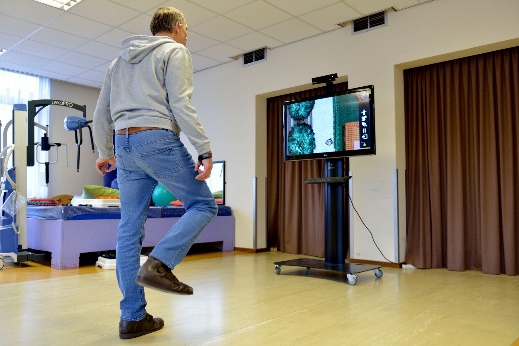 |
| Exergaming  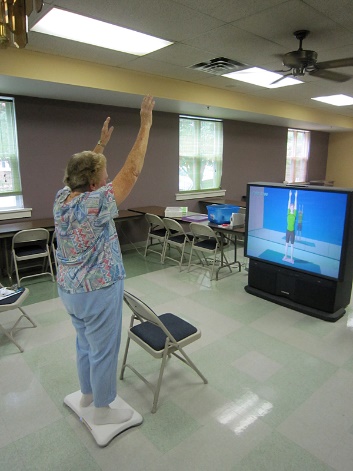 |  |

**Supplement 2: Focus group topic guide**

Exploring the use of eHealth

- What does eHealth for geriatric rehabilitation at home look like?
- What do/ or could you use?
- When would you choose to use eHealth?

*Participants wrote on flip-overs what their thoughts were on the following questions:*

Positive factors

- What goes well in the use of eHealth for the geriatric rehabilitation at home?
- Which promoting factors do you experience in relation to the implementation of eHealth in geriatric rehabilitation at home?

Negative factors

- What could be done better in the use of eHealth for geriatric rehabilitation at home?
- Which hindering factors do you experience in relation to the implementation of eHealth in geriatric rehabilitation at home?

*To receive the details in what the participants wrote down an explanation was asked for and to the entire group was asked how you could use or overcome the promoting/ hindering factors.*

Projection technique

*Each participant was asked to think of a type of eHealth that, according to them, would fit best for geriatric rehabilitation at home. They had to describe the user requirements. Their answers were discussed and elaborated upon.*

**Supplement 3: Full stakeholder table**

|  | N | Power^1^ | Legitimacy^2^ | Urgency^3^ | None | Don't know |
| --- | --- | --- | --- | --- | --- | --- |
| Patient | 21 | **71,4%** | **76,2%** | **52,4%** |  |  |
| Informal caregiver | 21 | **66,7%** | **71,4%** | 28,6% |  |  |
| Family | 21 | 47,6% | **57,1%** | 9,5% | 4,8% |  |
| Elderly care physician | 21 | 47,6% | 47,6% | 28,6% | 14,3% | 9,5% |
| Nurse practitioner | 21 | **52,4%** | 42,9% | 23,8% | 9,5% | 9,5% |
| Occupational therapist | 21 | **61,9%** | **66,7%** | 38,1% |  | 9,5% |
| Psychologist | 21 | 47,6% | 47,6% | 9,5% | 9,5% | 14,3% |
| Physiotherapist | 21 | **66,7%** | **71,4%** | 38,1% | 0,0% | 4,8% |
| Speech therapist | 21 | **57,1%** | 47,6% | 14,3% | 4,8% | 9,5% |
| Dietician | 21 | 47,6% | 38,1% | 19,0% | 9,5% | 19,0% |
| Social worker | 21 | 47,6% | 47,6% | 23,8% | 0,0% | 14,3% |
| Spiritual counselor | 21 | 19,0% | 38,1% |  | 23,8% | 19,0% |
| General practitioner | 21 | 47,6% | **57,1%** | 23,8% | 19,0% | 9,5% |
| Practice assistant general practitioner | 21 | 28,6% | 33,3% | 19,0% | 23,8% | 19,0% |
| Nurse (quality of care) | 20 | 40,0% | **50,0%** | 20,0% | 5,0% | 15,0% |
| Nurse (level 4)^4^ | 20 | **55,0%** | 40,0% | 25,0% | 10,0% | 15,0% |
| Nurse (level 3)^4^ | 18 | 44,4% | 33,3% | 22,2% | 22,2% | 16,7% |
| Nurse (level 2)^4^ | 20 | 30,0% | 30,0% | 5,0% | 20,0% | 25,0% |
| Home care | 20 | **65,0%** | **50,0%** | 30,0% |  | 10,0% |
| District nurse | 20 | **75,0%** | **50,0%** | 35,0% |  | 5,0% |
| Policy maker | 21 | 19,0% | 23,8% | 4,8% | 38,1% | 23,8% |
| Geriatric rehabilitation manager | 21 | 38,1% | 47,6% | 9,5% | 19,0% | 9,5% |
| Planner | 20 | 10,0% | 35,0% | 14,0% | 45,0% | 10,0% |
| Client service desk | 20 | 10,0% | 25,0% |  | 40,0% | 25,0% |
| Project leader: innovation | 21 | 38,1% | 33,3% | 19,0% | 28,6% | 9,5% |
| Client council | 21 | 23,8% | 42,9% | 19,0% | 33,3% | 19,0% |
| ICT department^5^ | 21 | 9,5% | **52,4%** | 23,8% | 28,6% | 9,5% |
| Liaison nurse | 21 | 14,3% | 38,1% | 14,3% | 28,6% | 14,3% |
| Healthcare insurance company | 20 | 45,0% | **50,0%** | 35,0% | 15,0% | 5,0% |
| Care office | 20 | 35,0% | 40,0% | 15,0% | 25,0% | 10,0% |
| National Ehealth Living Lab | 21 | 19,0% | 33,3% | 4,8% | 9,5% | 52,4% |
| Pharmacy | 21 |  | 23,8% | 4,8% | 47,6% | 23,8% |
| Local authority | 21 | 28,6% | 47,6% | 23,8% | 23,8% | 14,3% |
| Restaurant staff (in the rehabilitation center) | 21 |  | 19,0% | 9,5% | 42,9% | 33,3% |
| Home care store | 21 | 4,8% | 23,8% | 14,3% | 38,1% | 23,8% |
| Housing association | 21 | 14,3% | 33,3% | 14,3% | 33,3% | 19,0% |
| Centre for care indication | 21 | 14,3% | 23,8% | 19,0% | 33,3% | 28,6% |

1 Power is the level of influence a stakeholder has on home-based geriatric rehabilitation and the use of eHealth
2 Legitimacy is the level in which a stakeholder needs to be legally, morally, or contractually involved in home-based geriatric rehabilitation and the use of eHealth
3 Urgency is the priority of the stakeholder in home-based geriatric rehabilitation and the use of eHealth
4 In The Netherlands the nurses have different education levels and responsibilities therefore their education level is specified.5 ICT is the information and communication technology department.

**Supplement 4: Interview and focus group participants**

Interview participants

| Participant | Gender | Age | Marital status | Digital health skills |
| --- | --- | --- | --- | --- |
| A | Female | 76 | Widow | Experienced |
| B | Female | 81 | Married | Experienced |
| C | Female | 75 | Married | Average |
| D | Female | 66 | Widow | Experienced |
| E | Female | 80 | Married | Average |
| F | Male | 74 | Married | Experienced |
| G | Female | 70 | Unmarried | Experienced |
| H | Female | 86 | Widow | Beginner |

Focus group participants

| Participant | Profession | Experience (years) | Focus area |
| --- | --- | --- | --- |
| I | Physiotherapist | 24 | Orthopedics |
| J | Physiotherapist | 1 | Orthopedics |
| K | Speech therapist | 15 | Neurology/ psychogeriatrics |
| L | Elderly care physician | 10 | Psychogeriatrics |
| M | Speech therapist | 6 | Neurology/ psychogeriatrics |
| N | Occupational therapist | 24 | Neurology |
| O | Dietician | 4 | Neurology/ psychogeriatrics |

**Supplement 5: The foundation and attributes underlying each value**

| **Foundation** | **Attributes** | **Value** |
| --- | --- | --- |
| **Categories interviews:** Digital skills, trying eHealth, Digital use: motivation, digital use: frequency, digital use: functions, personal approach  **Desk research:** Pharos ^1^, WHO ^2^, Federatie Medisch Specialisten ^3^  **Categories focus groups:** Determine digital skills, personalized digital health  **Categories questionnaire:** Digital skills | Stakeholders think it is important that…   - The technology matches, or can be adapted to fit, the digital skill level of the patient. - The technology can be used by older adults who experience physical barriers of using eHealth, such as vision. | Fit with the digital skills and needs of the patient |
| **Categories interviews:** Combination face-to-face & digital, consultation with distance, monitoring  **Desk research:** Federatie Medisch Specialisten ^3^, Kraaijkamp ^4^  **Categories focus groups:** Blended is necessary, reduced travel time, digital skills of the healthcare professional  **Categories questionnaire:** Time efficient, online vs in person, therapeutic relationship, blended | - eHealth should never replace the face-to-face therapy. - eHealth can only be used when both parties agree. - You should be able to stay in touch with the healthcare professionals after discharge. | Blended care |
| **Categories interviews:** Personalized approach, motivation, personal factors  **Desk research:** Federatie Medisch Specialisten ^3^, Kraaijkamp ^4^  **Categories focus groups:** Personal approach, individual goals  **Categories questionnaire:** Review information, personal contact | - The technology can be adapted to specific wishes and therapeutic needs of each patient. - The technology shouldn’t diminish the social aspect of the therapy. - The technology can be used on different devices. | Personalized |
| **Categories interviews:** Wish to return, it should be safe, independence, monitoring  **Desk research:** Federatie Medisch Specialisten ^3^, Zorginstituut Nederland ^5^  **Categories focus groups:** Secure safety  **Categories questionnaire:** Control & correct | - eHealth should only be used when it is safe to be used independently in the home situation. | Safety at home |
| **Categories interviews:** Costs, borrow appliances  **Desk research:** NZA ^6^ Raad voor Volksgezondheid & Samenleving ^7^  **Categories focus groups:** Costs, Budget and time | - It should be low cost, or even paid for by the insurance company. - The organization has a licensing budget. | Affordability |
| **Categories interviews:** Control & support, social contacts, support/ help from others  **Categories focus groups:** Family support | - There is a help desk for technological problems. - They can stay in touch with other patients. - There is a support system in place. | Support |
| **Desk research:** Federatie Medisch Specialisten ^3^, Ministerie van Justitie en Veiligheid ^8^  **Categories focus groups:** Privacy | - The technology is compliant with the privacy legislation and data is stored safely. | Privacy |

1. Pharos. Checklist Toegankelijke Informatie - eHealth Toepassing, <https://checklisttoegankelijkeinfo.pharos.nl/checklist> (2022, accessed 09-02 2023).

2. World Health Organization. *Monitoring the implementation of digital health: an overview of selected national and international methodologies.* 2022. Copenhagen: WHO Regional Office for Europe.

3. Federatie Medisch Specialisten, Zorgverzekeraars Nederland, de Nederlandse Vereniging van Ziekenhuizen, et al. *Handreiking telemonitoring*. 2022.

4. Kraaijkamp JJM, van Dam van Isselt EF, Persoon A, et al. eHealth in Geriatric Rehabilitation: Systematic Review of Effectiveness, Feasibility, and Usability. *J Med Internet Res* 2021; 23. DOI: 10.2196/24015.

5. Zorginstituut Nederland. Geriatrische revalidatiezorg/ art. 2.5c Besluit zorgverzekering. Diemen2021.

6. Nederlandse Zorgautoriteit. *Wegwijzer bekostiging digitale zorg 2023*. 2023. PUC Open Data.

7. Raad voor Volksgezondheid & Samenleving. *De kunst van het innoveren - Tijd voor een maatschappelijk perspectief op zorginnovatie*. 2022. Den Haag: Raad voor Volksgezondheid en Samenleving.

8. Ministerie van Justitie en Veiligheid. Handleiding Algemene verordening gegevensbescherming. 2.0 ed. 2022.
